# Supplementary material for: Long-term T cell fitness and proliferation is driven by AMPK-dependent regulation of reactive oxygen species
Source: Sci Rep. 2020 Dec 10;10:21673. doi: 10.1038/s41598-020-78715-2 (PMC7728748; doi:10.1038/s41598-020-78715-2)
Supplement: Supplementary file 1 — Supplementary Information. [file 41598_2020_78715_MOESM1_ESM.pdf]

Long-term T cell fitness and proliferation is driven by AMPK-dependent regulation of oxygen reactive species

Anouk Lepez, Tiphène Pirnay, Sébastien Denanglaire, David Perez-Morga, Marjorie Vermeersch, Oberdan Leo and Fabienne Andris

Figures S1 to S14

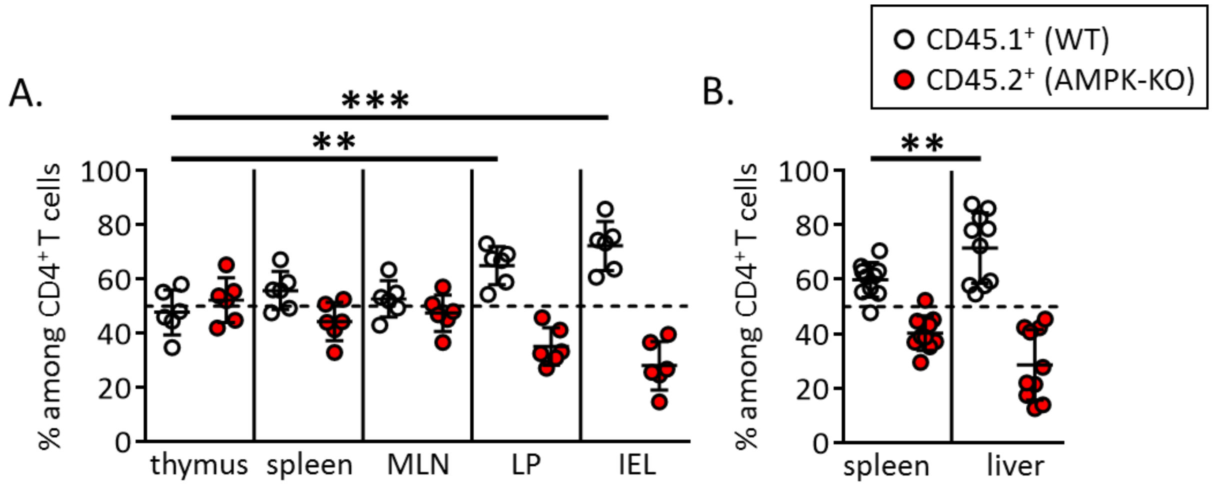

<u>Figure S1:</u> AMPK promotes peripheral T cells replenishment upon bone marrow transfer

(**A-B**) Relative contribution of AMPK-KO $CD4^+$ to the repopulation of the thymus, spleen, MLN, LP and IEL (**A**) of $CD3\varepsilon^{-/-}$ mice were reconstituted with a 1:1 mixture of bone marrow cells from WT (CD45.1) and $AMPK^{KO-T}$ (CD45.2) mice as in Figure 1 (symbols represent individual mice; 1 experiment representative of 3 with n = 6) and (**B**) spleen and liver (pool of 2 independent experiments with n = 10). Statistical analysis: Friedman followed by Dunn multiple comparison (**A**), Wilcoxon (**B**). ** $p < 0.01$; *** $p < 0.001$

MLN = Mesenteric lymph nodes, LP = Lamina propria, IEL = Intraepithelial lymphocytes

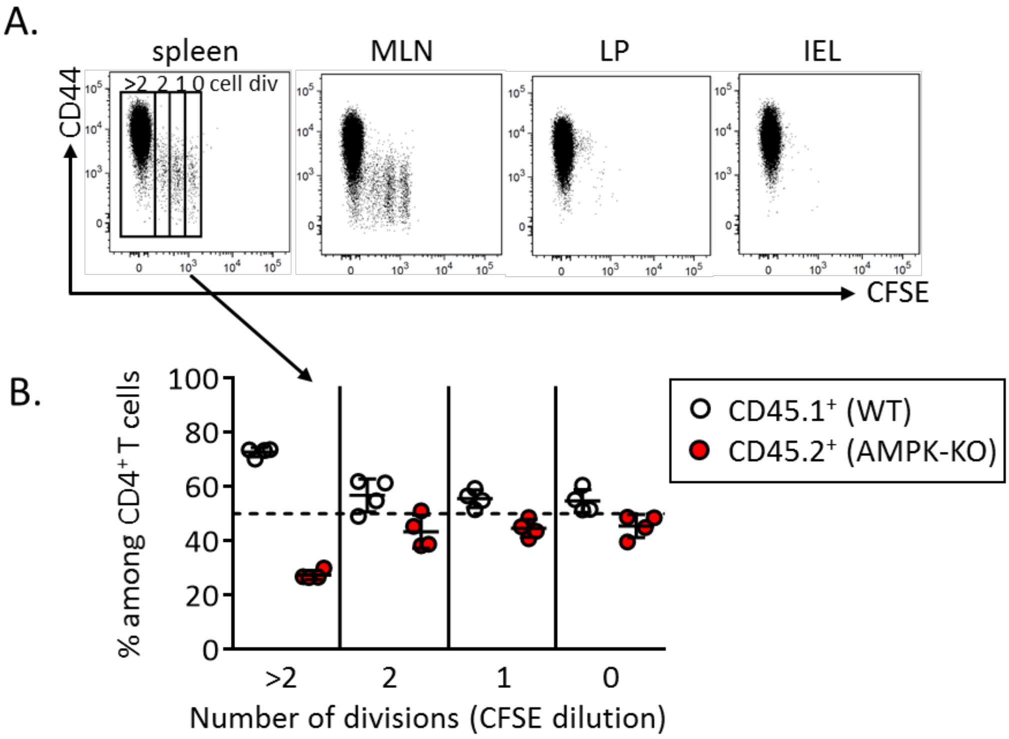

Figure S2: AMPK gradually promotes accumulation of divided cells during competitive homeostatic proliferation

CFSE-labeled WT (CD45.1) and AMPK$^{KO\text{-}T}$ (CD45.2) naive T cell (1:1 ratio) were i.v. injected into CD3ε$^{-/-}$ mice. Recovered WT and AMPK-KO T cells were analyzed by flow cytometry 10 days later.

(**A**) Expression of CD44 according to the number of cell divisions (CFSE dilution) in the spleen, MLN, LP and IEL of recipient mice. (**B**) Relative contribution of WT and AMPK-KO CD4^{+} T cells to the repopulation of the spleen according to the number of cell divisions (1 experiment representative of 3 with n = 4).

MLN = Mesenteric lymph nodes, LP = Lamina propria, IEL = Intraepithelial lymphocytes

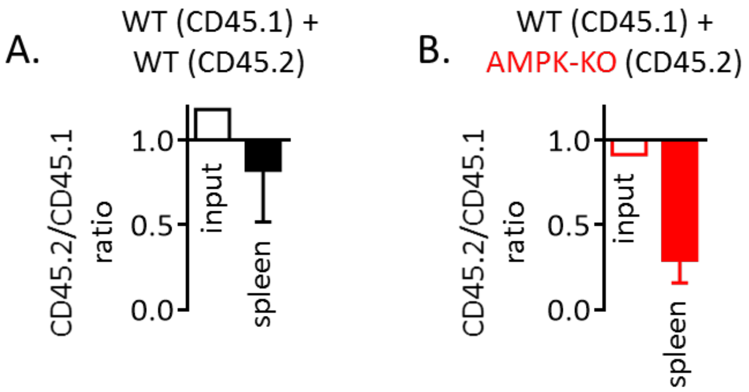

<u>Figure S3:</u> AMPK-KO T cell defect in homeostatic proliferation does not result from CD45 polymorphism

WT (CD45.1) naive T cells were mixed with mice WT (**A**) or AMPK-KO (**B**) counterparts (both from CD45.2 mice) and inoculated into CD3ε$^{-/-}$ mice. Results represent the relative contribution of CD45.2 cells in the inoculum (input) and in the spleen of recipient mice on day 10 after transfer (mean +/- SD of 3 recipient mice).

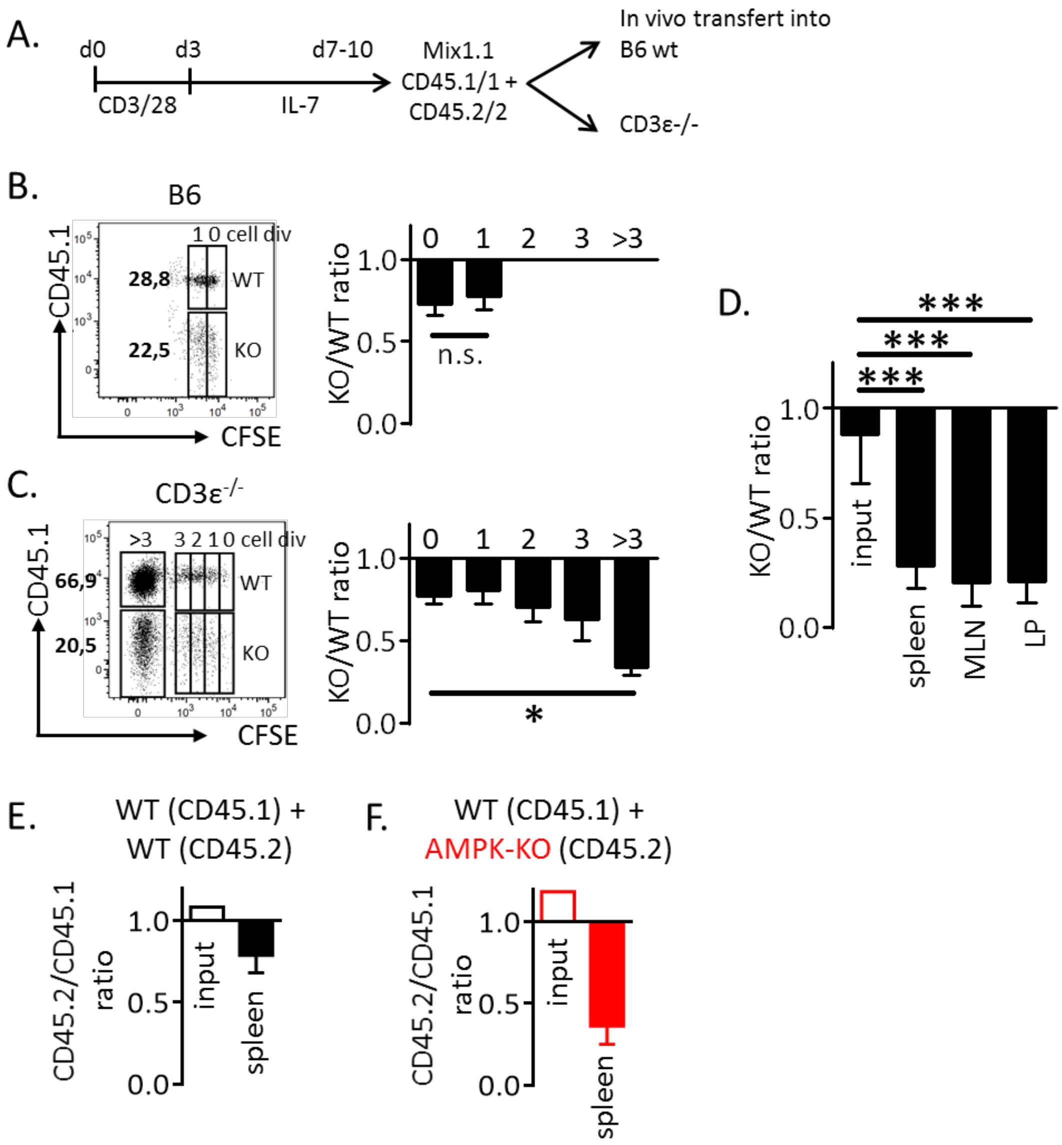

Figure S4: AMPK-KO memory-like Th cells exhibit impaired homeostatic proliferation

(**A-D**) IL-7-driven memory-like Th1 cells from WT (CD45.1) and AMPK^{KO-T} (CD45.2) mice were mixed at 1:1 ratio and injected i.v. into C57BL/6 (**B**) or CD3ε$^{-/-}$ (**C-D**) mice. Recovered T cells were analyzed on day 10 by flow cytometry. (**A**) Experimental protocol. (**B, C**) Dot plots and histograms show the relative proportions of WT and AMPK-KO cells in the spleen of recipient mice (CD4^{+} gated), according to CFSE division status. Numbers upper each box indicate cell division number, numbers on the left side indicate the percentage of cells in the corresponding gate. (**D**) Relative contribution of AMPK-KO memory Th1 cells to the repopulation of the CD4^{+} T cell subset in the spleen, MLN and LP of CD3ε$^{-/-}$ mice. Data are representative of 3 independent experiments with n = 5 (**B, C**) or pooled from 3 experiments, with n = 13 (**D**).

(**E-F**) Memory-like Th1 cells from WT (CD45.1) mice were mixed with WT (**E**) or AMPK-KO (**F**) (both CD45.2) counterparts and inoculated into CD3ε$^{-/-}$ mice. Results represent the relative contribution of CD45.2 cells in the inoculum (input) and in the spleen of recipient mice on day 10 after transfer (mean +/- SD of 3 recipient mice).

Statistical analysis: Wilcoxon (**B, D**), Friedman followed by Dunn multiple comparison (**C**). * p < 0,05, *** p < 0,001

MLN = Mesenteric lymph nodes, LP = Lamina propria

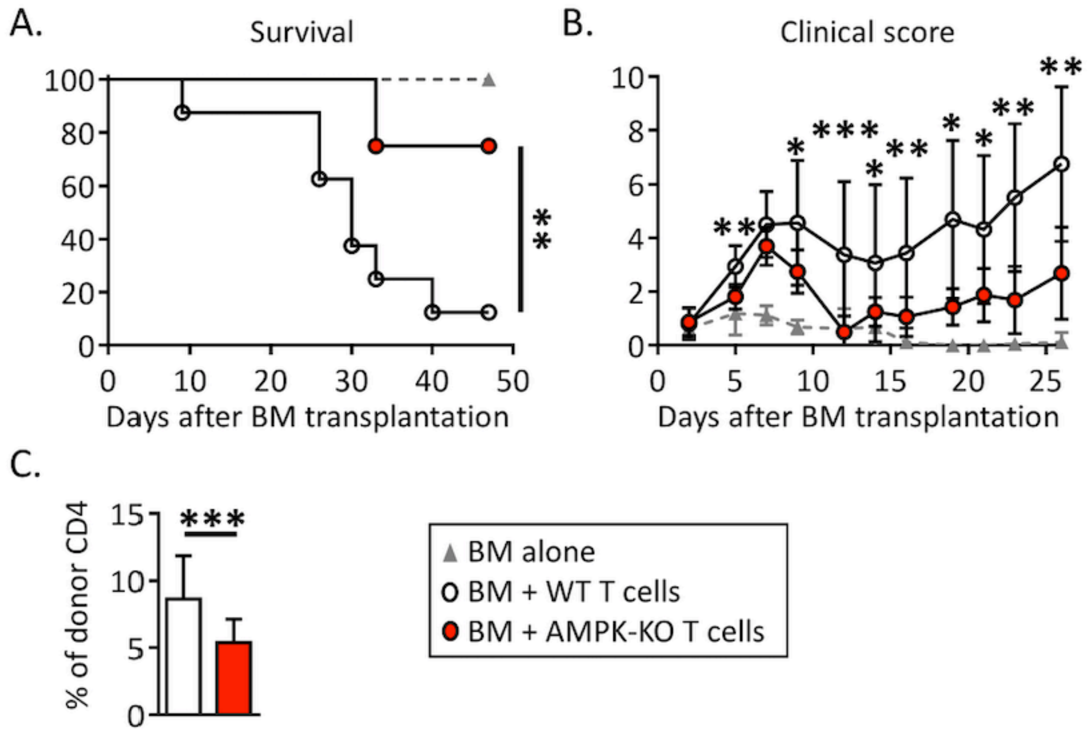

<u>Figure S5:</u> Knock-down of AMPK in donor T cells decreases the severity of GVHD

(**A, B**) Survival (**A**) and clinical score (**B**) of host Balb/c mice transplanted with allogeneic WT or AMPK-KO T cells (n = 8 per group, 1 experiment representative of 3). (**C**) Percentage of allogenic $CD4^+$ T cells in the spleen of the Balb/c host mice. Data are pooled from 5 independent experiments with a total n = 26 (WT group) and 27 (AMPK-KO group).

Statistical analysis: Mann–Whitney (**B, C**), Mantel-Cox (**A**). * $p < 0.05$; ** $p < 0.01$; *** $p < 0.001$

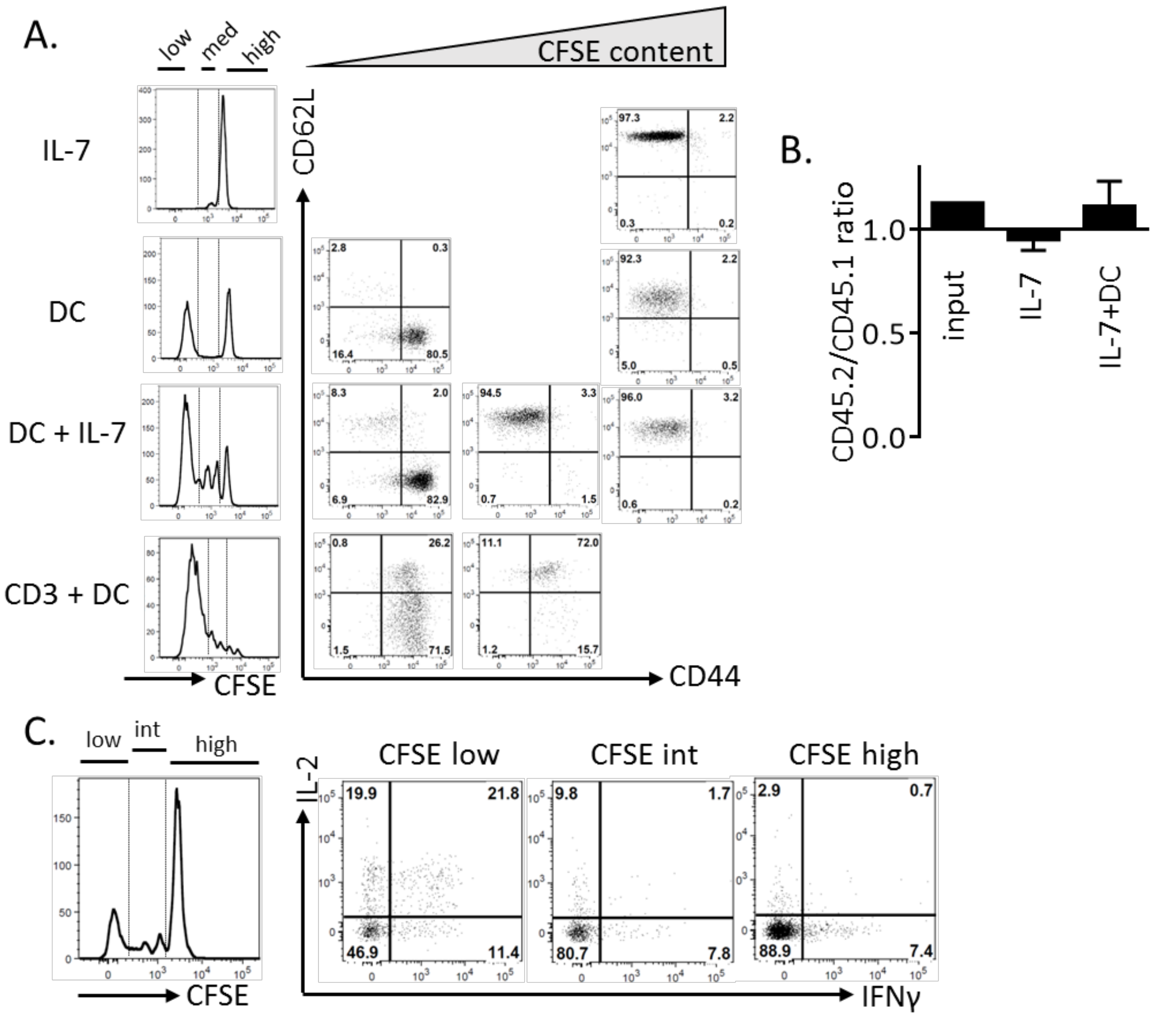

Figure S6: Model of IL-7 driven homeostatic proliferation *in vitro*

(**A**) Naive T cells from C57BL/6 mice were labeled with CFSE and cultured in the presence of IL-7 and / or syngeneic DCs, as indicated. Culture media were replaced with fresh DCs and IL-7 each 4-5 day and cells were analyzed on day 14 and 18. Additional control included cells activated in the presence of DCs + anti-CD3 mAbs for 5 days. CFSE dilution (left panels) and CD44 / CD62L expression (gate $CD4^+$) as a function of CFSE content. (**B**) Naïve T cells from WT (CD45.1) mice were mixed with WT (CD45.2) counterparts and cultured in the presence of IL-7 (n=8) or IL-7+syngeneic DCs (n=12). Results represent the relative contribution of CD45.2 cells in the inoculum (input) and after 13 days of culture.

(**C**) Naive T cells from C57BL/6 mice were labeled with CFSE and cultured in the presence of IL-7 and syngeneic DCs. Percentage of $CD4^+$ T cells producing IL-2 and IFNγ according to the number of cell divisions after 14 days of culture.

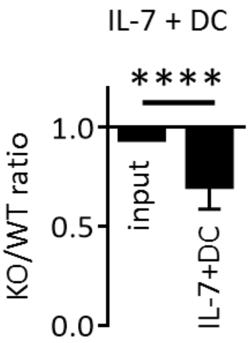

<u>Figure S7:</u> AMPK-KO memory-like Th cells exhibit impaired homeostatic proliferation *in vitro*

Relative recovery of AMPK-KO memory-like Th1 cells after a 9 day-homeostatic culture with IL-7 and syngeneic DCs.

Statistical analysis: Wilcoxon ; **** $p < 0.0001$

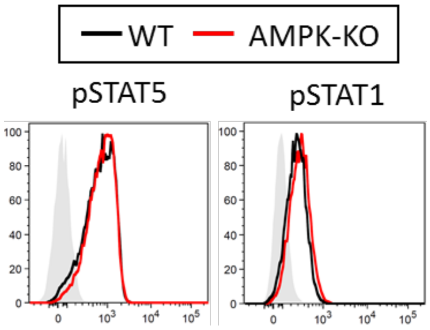

<u>Figure S8</u>: AMPK-KO Th cell express normal phospho-STAT5/STAT1 signaling in response to IL-7

Phosphorylation levels of STAT5 and STAT1 after 30 min of IL-7 treatment (data are representative of at least 3 experiments). Filled grey histograms represent unstained Th cells.

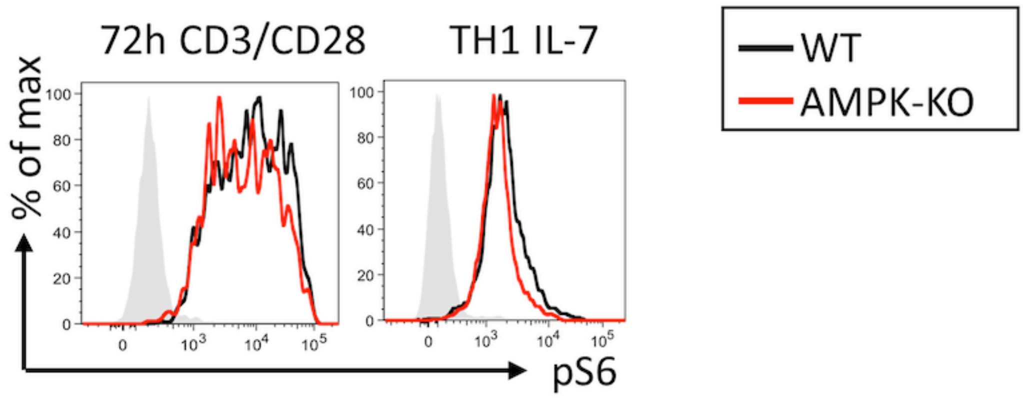

<u>Figure S9:</u> AMPK-KO Th cell express normal levels of phospho-S6

Phosphorylation levels of S6 ribosomal protein in anti-CD3/CD28-activated naive T cells or IL-7-driven memory Th1 cells (data are representative of 2 experiments). Filled grey histograms represent unstained Th cells.

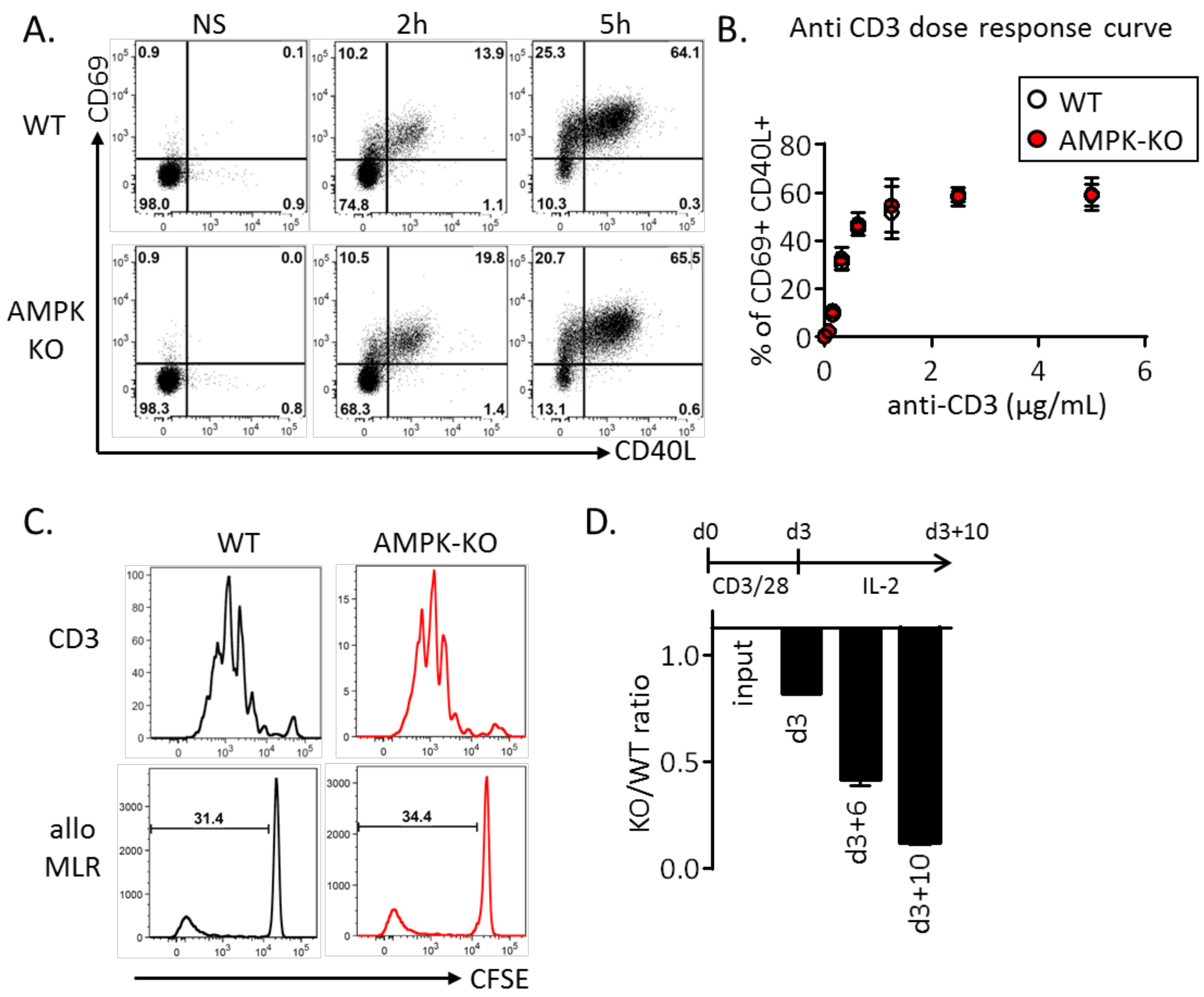

Figure S10: AMPK is not required for early naive T cell activation and proliferation but confers a competitive advantage during sustained T cell expansion

(**A**) Early acquisition of the activation markers CD69 (H1.2F3, eBioscience) and CD40L (MR1, eBioscience) by WT and AMPK-KO memory-like Th cells after 2 and 5h of anti-CD3/ CD28 stimulation (1 experiment representative of 3). (**B**) Dose response to anti-CD3 stimulation of WT and AMPK-KO memory-like Th cells after 2h (pool of 2 independent experiments, n=2). (**C**) CFSE dilution profiles of WT and AMPK-KO naive T cells stimulated with anti-CD3 / CD28 mAbs (upper panel) or Balb/c-derived allogeneic DCs (lower panel) during 5 days (1 experiment representative of 3). (**D**) Naive T cells were polarized into Th1 cells for 3 days with plate-bound anti-CD3, anti-CD28, IL-12 and anti-IL-4. Th1 cells were further diluted and cultured for an additional 10 days in IL-2-supplemented (20 ng/ml, Peprotech) media to obtain effector-like cells. Data express the relative recovery of AMPK-KO T cells after anti-CD3/ CD28 stimulation (day 3) and after 6 and 10 day of IL-2-supplemented culture (day 3+6 and 3+10, respectively). 1 experiment representative of 2, with n = 2 (day 3) or n = 5 (day 3+6 and day 3+10).

NS = unstimulated

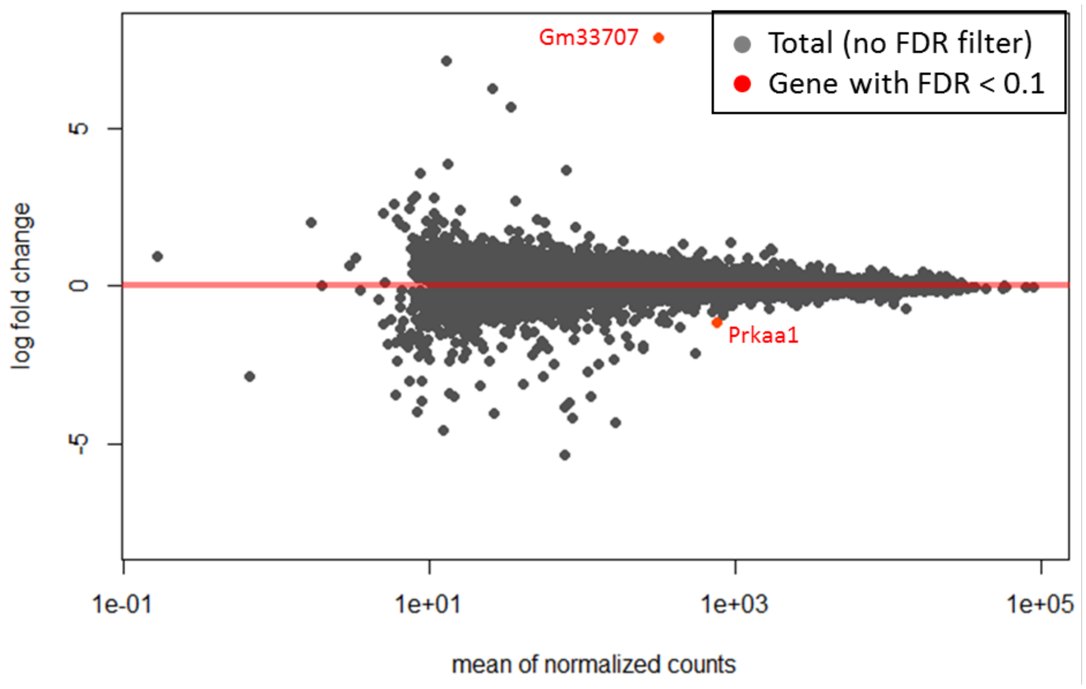

<u>Figure S11</u>: Homeostatic proliferating WT and AMPK-KO T helper cells express a similar gene pattern

WT and AMPK-KO naïve T cells were recovered after of 14 days of HP with IL-7 and syngeneic DCs and subjected to RNAseq analysis (1 experiment, n = 3 per group). (**A**) MA of the differentially expressed genes (DEGs) with low expression genes (less than 20 read in WT) excluded. Y-axis: logFC (fold change); X-axis: average expression for each gene; the color of the data points denotes the status of DEGs (red points: FDR <0.1; grey points: with FDR >0.1). CPM, counts per million; FDR, false discovery rate. Only two transcripts: Gm33707 and Prkaa1 show distinct expression between WT and AMPK-KO (FDR < 0.1).

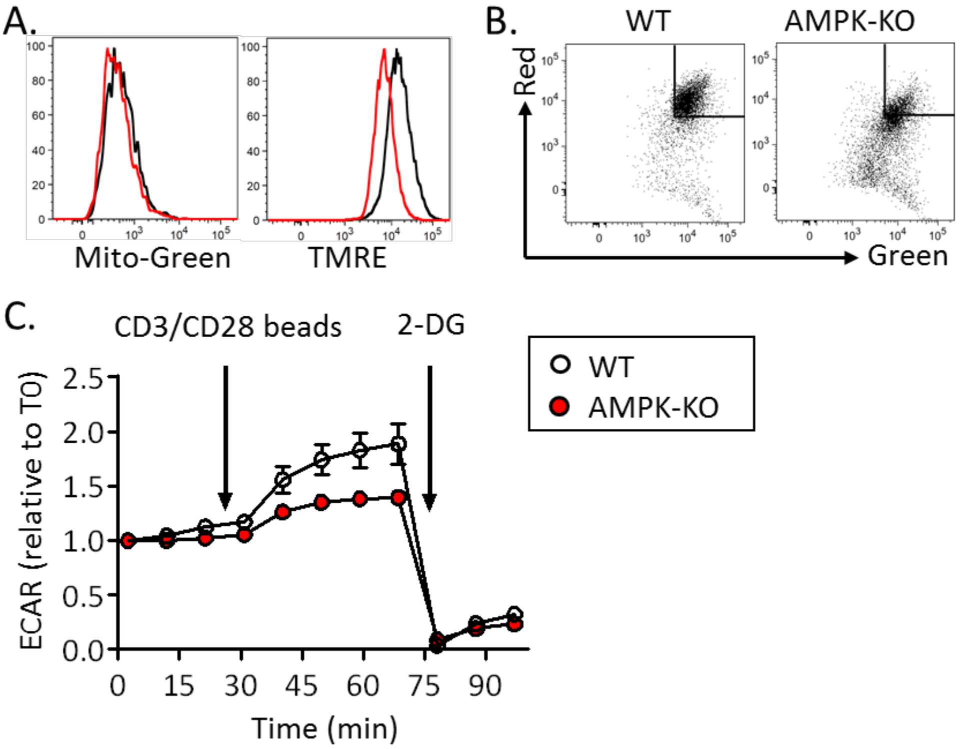

<u>Figure S12</u>: Defective mitochondrial stress-induced glycolytic switch in AMPK-KO memory-like Th cells

Naive CD4^{+} T cells from WT or AMPK$^{KO\text{-}T}$ mice were polarized to memory-like Th1 cells by a 3-day stimulation in Th1 condition followed by an additional 4-day culture in IL-7-supplemented media.

(**A**) Mitochondrial mass (Mito-Green) and (**B**) mitochondrial membrane potential (TMRE) in WT and AMPK-KO memory-like Th1 cells. Data are representative of 3 independent experiments.

(**C**) ECAR measurement after treatment of memory-like Th1 cells with anti-CD3/CD28-coated beads as determined by Seahorse experiment. Data are representative of 2 independent experiments with n = 3.

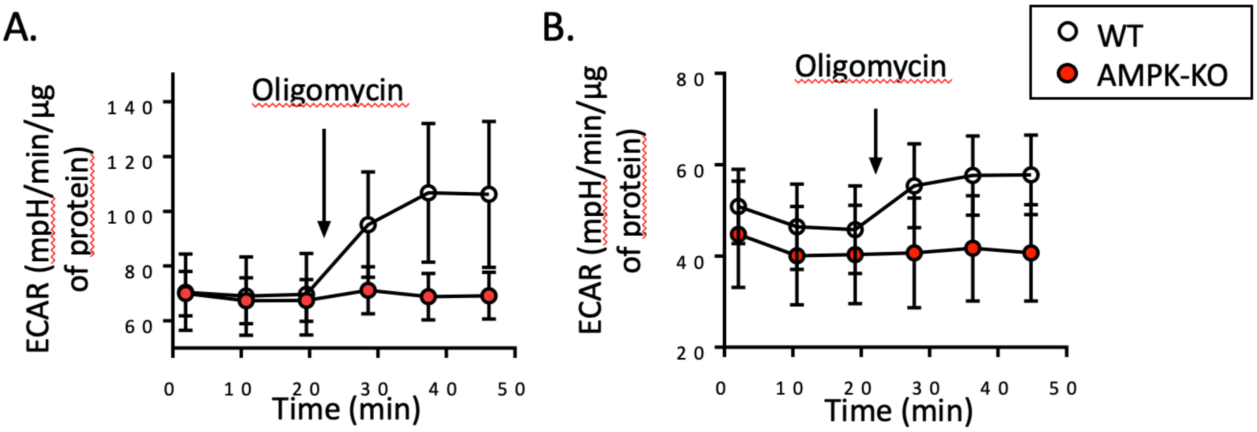

<u>Figure S13</u>: Defective glycolytic switch in AMPK-KO T cells upon mitochondrial stress

Kinetic of ECAR upon oligomycin treatment of WT and AMPK-KO: raw data from Figure 5

(A) IL-7+DC-expanded T cells
(B) Th1 memory-like cells

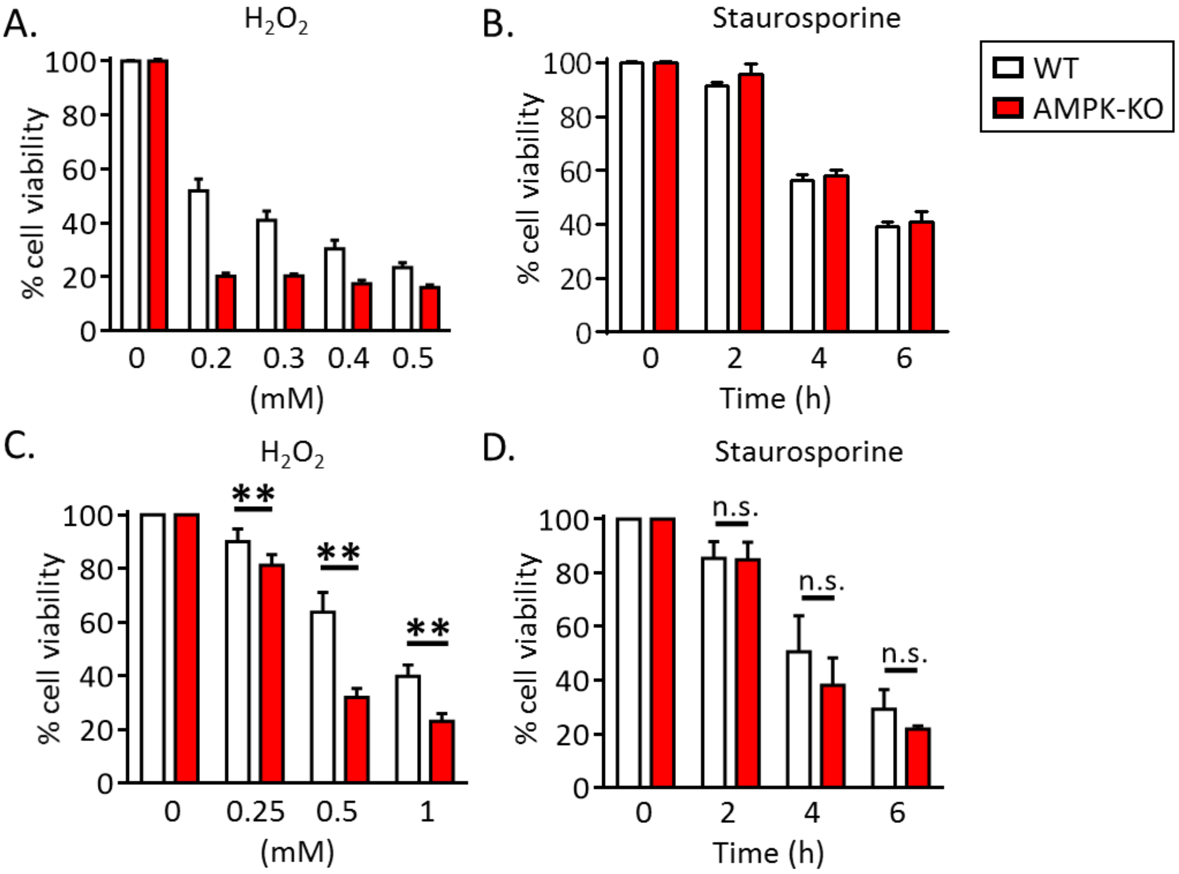

<u>Figure S14</u>: AMPK dampens ROS toxicity

Percentages of naïve T cell (**A, B**) or memory-like Th1 cell (**C, D**) viability after 2h-treatement with graded doses of H_2O_2 (**A, C**) or after treatment with staurosporine (5 µM) (**B, D**). Results are representative of 5 individual experiments (n = 2) or pooled from 4 (n = 4), 2 (n = 6) and 3 (n = 3) independent experiments (**B**, **C**, **D** panels, respectively).

Statistical analysis: Mann-Whitney (**B- D**). ** p < 0,01
